# Reacquisition of cocaine conditioned place preference and its inhibition by previous social interaction: Neurochemical and electrophysiological correlates in the nucleus accumbens corridor

**DOI:** 10.1186/2193-1801-4-S1-L2

**Published:** 2015-06-12

**Authors:** Alois Saria, Janine Prast, Aurelia Schardl, Kai Kummer

**Affiliations:** Experimental Psychiatry Unit, Medical University Innsbruck, Austria

**Keywords:** Addiction, medium spiny neurons, behaviour

The nucleus accumbens has long been a major target for studies on the rewarding effects of drugs of abuse or physiological reinforcers, whereas the brain regions medial of the medial accumbens shell have received less attention. We investigated if counterconditioning with dyadic (i.e., one-to-one) social interaction, a strong inhibitor of the subsequent reacquisition of cocaine conditioned place preference (CPP), differentially modulates the activity of the diverse brain regions oriented along a mediolateral corridor reaching from the interhemispheric sulcus to the anterior commissure, i.e., the nucleus of the vertical limb of the diagonal band, the medial septal nucleus, the major island of Calleja, the intermediate part of the lateral septal nucleus, and the medial accumbens shell and core. EGR1 activation was predominantly found in dynorphin-labeled cells, i.e., presumably D1 receptor-expressing medium spiny neurons (D1-MSNs), with D2-MSNs (immunolabeled with an anti-DRD2 antibody) being less affected. Cholinergic interneurons or GABAergic interneurons positive for parvalbumin, neuropeptide Y or calretinin were not involved in these CPP-related EGR1 changes. Glial cells did not show any EGR1 expression either. Cocaine conditioning increased the spike frequency of neurons in the septal nuclei, whereas social interaction conditioning increased the spike frequency in the nucleus accumbens compared to saline control animals. In addition, social interaction conditioning decreased the amount of active neuron clusters in the nucleus accumbens. The present findings could be of relevance for the therapy of impaired social interaction in substance use disorders, depression, psychosis, and autism spectrum disorders.

